# Advances in artificial intelligence and computer science for computer-aided diagnosis of colorectal polyps: current status

**DOI:** 10.1055/a-2098-1999

**Published:** 2023-08-16

**Authors:** Querijn N E van Bokhorst, Britt B S L Houwen, Yark Hazewinkel, Paul Fockens, Evelien Dekker

**Affiliations:** 1 26066 Department of Gastroenterology and Hepatology, Amsterdam University Medical Centers, location Academic Medical Center, Amsterdam, the Netherlands; 2 571165 Amsterdam Gastroenterology Endocrinology Metabolism, Amsterdam, the Netherlands; 3 3913 Department of Gastroenterology and Hepatology, Tergooi Medical Center, Hilversum, the Netherlands

## Introduction

Colorectal cancer (CRC) is the third most commonly diagnosed malignancy and the second leading cause of cancer related-death in the world 1 . CRC develops from precancerous polyps through several (epi)genetic pathways 2 . Colonoscopy is considered the gold standard for detection and diagnosis of CRC and its precursor lesions 3 4 . Moreover, colonoscopy provides opportunities for endoscopic resection of precancerous polyps, which is known to be effective to prevent CRC 5 .

Despite reported benefits, colonoscopy outcomes may differ depending on quality of the endoscopist performing the procedure. Among others, this relates to differences in ability of endoscopists to accurately assess polyp characteristics such as location 6 , size 7 8 9 10 and morphology 11 12 13 , as well as differences in their performance in predicting histological polyp features (e.g. histological subtype 14 15 16 , grade of dysplasia 17 18 19 , and, in case of suspected malignancy, presence of deep submucosal invasion [DSI] 20 21 22 ). These polyp characteristics are essential to decide on the indication for resection and histopathological analysis 23 24 , appropriate resection method 25 26 and appropriate surveillance interval 27 28 . Hence, inaccurate endoscopic polyp assessment could lead to higher patient and economic burden due to unnecessary polyp resection and analysis, as well as suboptimal treatment and/or surveillance regimens.

Over the last decade, artificial intelligence (AI) in biomedical science has received growing attention. AI can be defined as the simulation of human intelligence by computer systems 29 . Specific AI techniques such as machine learning can be used to make machines (computers) smarter through experience-based learning 30 31 . Since computer systems can be trained with a large amount of high quality and expert-annotated data, they could possibly serve as an objective, real-time, expert-level second observer modality during colonoscopy procedures. This might provide opportunities to reduce interobserver variability and improve general polyp assessment accuracy.

While evidence is currently scattered, we aimed to write a narrative review to provide a broad overview of current developments within the field of AI and computer science for computer-aided assessment of colorectal polyps. This includes assessment of polyp location, size, morphology and histology, including degree of dysplasia (low grade dysplasia [LGD] versus high grade dysplasia [HGD]) and, in case of suspected malignancy, invasion depth. Since computer-aided polyp detection concerns an already more thoroughly studied and evaluated topic 32 33 , developments within this field will not be addressed within this review.

## Methods

A comprehensive literature search was performed in the MEDLINE/PubMed, Embase and Cochrane Libraries from the inception of the databases up to and including the 17 ^th^ of July 2022. Key search terms used were “colorectal,” “polyp,” “artificial intelligence,” “size,” “location,” “morphology,” “histology,” “dysplasia” and “invasion depth.” Only studies published in English were screened. Reference lists of retrieved studies were manually screened to identify other relevant publications.

## Results

### Computer-aided assessment of polyp location

Accurate determination of polyp location is important to facilitate identification of a polyp or polypectomy site during consecutive colonoscopies and/or surgical procedures. In addition, polyp location can aid in polyp histology prediction 34 and is important to adopt the ‘leave-in-situ’ optical diagnosis strategy in daily practice 23 24 .

To determine the location of the endoscope tip during colonoscopy procedures, and hence the location of observed polyps, endoscopists often rely on identification of various endoscopic anatomical landmarks and differences in colonic caliber, color tones and vasculature of different colon segments 35 . Endoscope intubation depth in centimeters could also be used. However, due variations in colon length, shape and anatomy 36 37 38 , change in colon length and position due to insufflation and endoscope intubation, and curving and bending of the endoscope due to the colon’s flexibility and elasticity, the accuracy of these methods seems limited. This is illustrated by earlier studies describing considerable interobserver variability 6 and 18% to 34% incorrect endoscopic localization of colorectal lesions when compared to findings during consecutive surgical procedures 39 40 41 42 43 44 .

Several deep learning approaches for orientation in the colon based on analysis of endoscopic videos and images have been proposed ( Table 1 ) 45 46 47 . Two studies described deep learning approaches for either recognition of anatomical landmarks 45 or distinguishing different colon segments 46 (accuracies 66.6% to 92.0%). Another study described several camera localization approaches, among which the localization approach based on analysis of camera motion in between colonoscopy video frames reached highest accuracy (71.8% in test set) 47 .

**Table:** **Table 1** Overview of studies describing deep learning approaches for determination of location within the colon based on analysis of endoscopic videos and images.

	Year	Described approach	Classification groups	Datasets*	Results Accuracy (%)
Che et al. 45	2021	Deep learning model for recognition of endoscopic anatomical landmarks within video-derived colonoscopy images	Hepatic flexure Splenic flexure SDCJ	Training set: 6,911 images Test set: 1,729 images	90.7–92.0†
Saito et al. 46	2021	Deep learning model for distinguishing endoscopic colorectal images captured within different segments of the colon	Terminal ileum Cecum ACTTC DCTSC Rectum Anus	Training set: 9,995 images Test set: 5,121 images	66.6
Yao et al. 47	2021	Deep learning model for estimation of relative location of the endoscope camera within the colon based on (analysis of) camera motion in between video frames‡	Cecum Ascending colon Transverse colon Descending colon Sigmoid Rectum	Training set: 13 videos Test set: 3 videos	71.8
SDCJ, sigmoid-descending colon junction; ACTTC, ascending colon to transverse colon; DCTSC, descending colon to sigmoid colon *Data used for internal validation is reported as part of the training set. †After post-processing through identification of incorrectly predicted frames (based on their temporal distribution) and reassigning these frames to the correct class, accuracies increased up to 99.8%. ‡Results for other methods (based on withdrawal time analysis, based on endoscope imaging device) not reported due to inferior results.					

Proposed systems could possibly aid endoscopists in orientation within the colon. However, current studies still concern feasibility studies and accuracy is mostly still limited. Besides, usage of a segment classification that assumes that all colons and segments are of similar length currently limits feasibility of the proposed motion-based localization system 47 . In addition, the issue concerning the lack of a solid reference standard should be addressed. While mostly only estimation of position within the colon by the endoscopist is available as reference standard, some sort of bias concerning training and (clinical) validation of such systems will likely always be present.

Toward the future, the issue of a lack of a solid reference standard could possibly be addressed by using magnetic endoscopic imaging (MEI) devices. These devices can improve accuracy of determination of location within the colon during colonoscopy 39 48 49 50 . However, performance with aid of MEI devices is also not flawless and large-scale clinical trials assessing specific benefits of these devices for improving accuracy of polyp localization are still scarce. Thus, there is a need for further optimization and validation of MEI-assisted localization approaches, which may also improve the feasibility of existing deep learning approaches based on MEI data and images 47 51 . Simultaneously, composition of more robust datasets for algorithm training, preferably only containing images/videos that are annotated by multiple experts, could aid in creating a more reliable reference standard. Variability in colon length could possibly be assessed, and accounted for, by using recently developed applications for image depth estimation and topographical reconstruction 52 53 54 , assessment of endoscope camera pose 55 and endoscopic three-dimensional (3D) colon reconstruction 56 57 58 59 . Besides, 3D colon reconstruction 56 57 58 59 techniques might open doors for development of other polyp localization approaches, since these could potentially visualize detected polyps within reconstructions of the complete colon.

### Computer-aided assessment of polyp size

Polyp size has been shown to be associated with the risk that a polyp harbors advanced histological features 60 , as well as the risk of metachronous advanced lesions and CRC 27 28 . Hence, recommendations for appropriate resection method 25 26 and surveillance intervals 27 28 are determined, among other factors, by polyp size. Besides, polyp size determines whether a polyp can be included in the 'leave-in-situ' and 'resect-and-discard' optical diagnosis strategies for diminutive (1 to 5 mm) polyps 23 24 .

In daily practice, polyp size is based on visual estimation by the endoscopist. However, this strategy is prone to interobserver variability 7 8 9 10 , resulting in 10% to 35% inappropriate surveillance recommendations 9 10 . To reduce interobserver variability, methods for automated polyp size measurement using deep learning approaches 61 62 63 64 65 and computer vision techniques 64 66 have been proposed ( Table 2 ). Reported accuracies within these studies ranged between 79.2% to 88.0% 61 62 64 . Two studies benchmarking the performance of computer systems against that of endoscopists showed that computer systems may reach superior accuracy 64 65 .

**Table:** **Table 2** Overview of studies describing deep learning approaches or computer vision techniques for endoscopic polyp size measurement.

	Year	Described approach or technique	Classification groups	Dataset(s*	Size ground truth	Endoscopist comparison group (experience)	Measurement method comparison group	Results	
								CADx (accuracy %)	Endoscopists (accuracy %)
Chadebecq et al. 66	2015	Detection of Infocus-Breakpoint	Exact size estimation	Training set: 15 colonoscopy videos Test set: 5 colonoscopy videos	Visual estimation endoscopists (surgical tool as reference)	N/A	N/A	N/A ^†^	N/A
Itoh et al. 61	2018	Deep learning model	Binary approach: ≤ 10 mm vs. ≥ 10 mm	Training set: 34,396 images Test set: 13,093 images	Unspecified	N/A	N/A	79.2	N/A
Itoh et al. 62	2021	Deep learning model	Binary approach: ≤ 10 mm vs. ≥ 10 mm	Training set: 94,980 images Test set: 15,569 images	Measurement with sheath of polypectomy snare as reference (consensus of 3 experts)	N/A	N/A	81.0–88.0	N/A
Su et al. 63	2021	Deep learning model	Exact size estimation	Training set: N/A Test set: N/A	Pre-measured balls used for model development	N/A	N/A	N/A ^‡^	N/A
Abdelrahim et al. 64	2022	Photogrammetric imaging (structure from motion) technique	Binary approach: ≤ 5 mm vs. ≥ 5 mm	Training set: not reported Test set: 22 videos	Phantom polyps of known size	10 endoscopists (varying degree of experience)	Visual estimation	85.2	59.9 ^§^
		Deep learning model	Binary approach: ≤ 5 mm vs. ≥ 5 mm	Training set: 219 videos Test set: 10 videos	Visual size estimation endoscopists (mean of 3 experts)	N/A	N/A	80.0	N/A
Kwak et al. 65	2022	Deep learning model	Exact size estimation	Training set: N/A ^¶^ Test set: 90 images	Measurement with ruler after resection	4 experts (> 10,000 colonoscopies), 4 trainees (< 200 colonoscopies)	Visual estimation, opened snare measurement	N/A ^††^	N/A ^‡‡,§§^
CADx, computer-aided diagnosis; N/A, not available. * Data used for internal validation reported as part of the training set. ^†^ Instead of accuracy, mean error from ground truth reported: 4.5% to 6.4% (≈0.2 to 0.3 mm). ^‡^ Study described the process of model development for polyp size estimation. No specific results in terms of accuracy, sensitivity, specificity, negative predictive value, and positive predictive value reported. ^§^ Significant differences compared to CADx performance (P < 0.05). ^¶^ Model was built based on four datasets that are widely used for retinal vascular segmentation research. No specific polyp images were used for training. ^††^ Instead of accuracy concordance correlation coefficient (CCC) reported: 0.961. ^‡‡^ Instead of accuracy concordance correlation coefficient (CCC) reported: for visual size estimation CCC ranged between 0.650 and 0.758 (experts) and 0.465 and 0.703 (trainees). For open biopsy forceps size estimation CCC ranged between 0.789 and 0.815 (experts) and 0.657 and 0.762 (trainees). ^§§^ For visual size estimation significant difference reported for all endoscopists. For open biopsy forceps measurement significant differences reported for all but one expert endoscopist.									

While most studies showed promising results, some issues should be addressed. Most importantly, similar to polyp location, a robust reference standard is not available for polyp size. This is illustrated by the fact that different reference standards were used in the different studies, limiting robustness and comparison of performance of the different systems. Additionally, several studies used binary polyp size classifications 61 62 64 . Use of binary approaches hampers reliable comparison to systems using exact polyp size estimation approaches.

Next steps for the development of more robust computerized polyp size measurement methods should include prospective evaluation of proposed systems in real-time clinical settings. Simultaneously, the problem concerning the lack of a solid reference standard might possibly be addressed through usage of recently developed endoscope-integrated or -attached polyp measurement tools 67 68 69 . Although it is unlikely that these tools will facilitate determination of the true size of polyps without a certain margin of error, as can only be accomplished by measuring polyps in colon (segment) resection specimens, they could possibly aid in obtaining highly reliable estimates of polyp size within in vivo settings. This relates to the fact that these tools can be precisely calibrated and validated using (artificial) polyps of known size in ex vivo settings. However, in order to gain further insights into feasibility of these tools, large-scale clinical studies validating accuracy of these tools are still required.

### Computer-aided assessment of polyp morphology

Polyp morphology is an important feature for polyp malignancy risk-assessment 70 and can aid endoscopists in prediction of presence of DSI 20 21 71 72 . As such, morphology also aids in selecting the optimal resection method 25 26 . Assessment of polyp morphology is usually performed based on the Paris classification system 73 or laterally spreading tumor classification 74 , but accuracy is known to be observer-dependent 11 12 13 . Despite these facts, computer-aided diagnosis of polyp morphology is a field yet to be explored: to the best of our knowledge, only one study describing assessment of polyp morphology by a computer system, as part of an algorithm for automated textual polyp image description, is available 75 .

## Computer-aided prediction of polyp histology

### Differentiation of diminutive neoplastic from non-neoplastic (or adenomatous from non-adenomatous) polyps

Colorectal polyps can generally be subdivided into neoplastic and non-neoplastic. Neoplastic lesions concerns both lesions yielding malignant potential and malignant lesions, while non-neoplastic lesions do not yield malignant potential. Hence, removal and analysis of non-neoplastic lesions is often unnecessary 23 24 . Real-time optical differentiation of neoplastic and non-neoplastic polyps during colonoscopy procedures could help to reduce the significant patient and economic burden caused by unnecessary resection and analysis of non-neoplastic lesions 76 . For this reason, the 'leave-in-situ' and 'resect-and-discard' optical diagnosis strategies have been proposed 23 24 . However, while proposed optical diagnosis performance thresholds are frequently not met in community practice, feasibility of these strategies is still limited 14 15 16 .

A wide variety of studies describing computer systems trained to differentiate neoplastic and non-neoplastic lesions based on polyp phenotype has been published. For this review, we will highlight available prospective clinical trials evaluating the performance of such systems in real-time clinical settings and using either white light, (magnified) narrow band imaging (NBI) or blue light imaging (BLI) imaging modalities ( Table 3 ) 77 78 79 80 81 82 83 84 . In these studies, overall accuracies of computer-aided diagnosis (CADx) systems ranged between 78.8% and 93.2% 77 78 80 81 82 83 . Reported accuracies for diminutive polyps located within the rectosigmoid ranged between 75.2% and 94.4% 78 81 82 84 . Within five studies, CADx system performance was benchmarked to performance by endoscopists 80 81 82 83 84 . Two studies reported significant differences, both in favor of the endoscopists and the CADx system 80 82 . Four studies reported performance of endoscopists with real-time assistance of a CADx system 79 81 82 83 84 . While no significant benefits were reported for computer-aided colonoscopy when compared to endoscopists alone, one of these studies did show that non-experts can eventually meet expert accuracy levels when performing real-time computer-aided polyp assessment on a regular basis 84 .

**Table:** **Table 3** Overview of prospective clinical trials assessing performance of deep learning-based computer-aided diagnosis systems for endoscopic differentiation between neoplastic and non-neoplastic polyps.

	Year	Multicenter	Classification groups	Imaging modality	Polyps assessed for clinical validation	Endoscopist comparison group (experience)		Results					
								CADx†		Endoscopists		CADx-assisted†	
								All	RS	All	RS	All	RS
Kominami et al. 77	2016	No	Adenomas vs. non-adenomas (SSLs excluded)	Magnified NBI	88 diminutive polyps (45 AD, 43 NAD) of which 54 in RS	N/A	Success rate CADx (%) HC diagnosis (%) Number of polyps Accuracy (%) Sensitivity (%) Specificity (%) PPV (%) NPV (%)	N/A N/A 88 93.2 93.0 93.3 93.0 93.3	N/A	N/A‡	N/A	N/A	N/A
Mori et al. 78 ^§^	2018	No	Adenomas vs. non-adenomas (SSLs excluded)	Endocytoscopy with NBI	466 diminutive polyps (287 AD, 175 NAD) of which 250 in RS	N/A ^¶^	Success rate CADx (%) HC diagnosis (%) Number of polyps Accuracy (%) Sensitivity (%) Specificity (%) PPV (%) NPV (%)	98.1 N/A 466 91.6 92.7 89.8 93.7 88.3	N/A N/A 250 94.4 93.3 95.2 93.3 95.2	N/A	N/A	N/A	N/A
Barua et al. 79	2022	Yes	Neoplastic (including SSLs for primary analyses) vs. non-neoplastic (including SSLs for secondary analyses)	(Ultra)-magnified NBI	892 diminutive polyps (359 NP, 533 NNP), all in RS	22 endoscopists (all 1–5 years of colonoscopy experience or performed 200–1000 colonoscopies)	Success rate CADx (%) HC diagnosis (%) ^††^ Number of polyps Accuracy (%) Sensitivity (%) Specificity (%) PPV (%) NPV (%)	N/A	N/A	N/A	N/A 74.2 892 N/A 88.4 83.1 78.9 91.5	N/A	N/A 92.6 892 N/A 90.4 85.9 82.0 92.8
Garcia-Rodgríguez et al. 80	2022	No	Adenomas vs. non-adenomas (including SSLs)	WLE	52 diminutive polyps (35 AD, 18 NAD) of which unspecified amount in RS	4 endoscopists (all staff endoscopists without specific training in optical diagnosis)	Success rate CADx (%) HC diagnosis (%) ^‡‡^ Number of polyps Accuracy (%) Sensitivity (%) Specificity (%) PPV (%) NPV (%)	N/A N/A 52 78.8 88.2 61.1 81.1 73.3	N/A	N/A N/A 52 71.1 58.5* 94.4* 95.2 54.8	N/A	N/A	N/A
Hassan et al. 81 ^§§^	2022	No	Adenomas vs. non-adenomas (including SSLs)	WLE ^§§^	476 diminutive polyps (163 AD, 291 NAD) of which 295 in RS	4 endoscopists (all > 2000 screening colonoscopies, training in optical diagnosis)	Success rate CADx (%) HC diagnosis (%) Number of polyps Accuracy (%) Sensitivity (%) Specificity (%) PPV (%) NPV (%)	95.4 N/A 454 86.8 82.0 89.5 81.0 90.1	98.6 N/A 291 91.8 82.0 93.2 65.3 97.6	N/A	N/A	–– 92.2 439 90.7 80.6 95.5 89.2 91.3	N/A 94.6 279 96.1 81.2 98.0 83.9 97.6
Houwen et al. 82	2023	Yes	Neoplastic (including SSLs) vs. non-neoplastic	NBI	429 diminutive polyps (300 AD, 41 SSLs, 82 HPs) of which 122 in RS	20 endoscopists (all BCSP certified)	Success rate CADx (%) HC diagnosis (%) ^¶¶^ Number of polyps Accuracy (%) Sensitivity (%) Specificity (%) PPV (%) NPV (%)	98.3 99.7 422 79.6 89.4 38.3 85.9 46.3	N/A 99.2 121 75.2 91.7 37.8 77.0 66.7	N/A 86.8 367 85.8 94.7 47.1 88.7 66.7	N/A 85.2 104 86.9 91.9 73.3* 89.5 78.6	N/A 98.1 415 84.1 93.2 44.9 88.8 60.3	N/A 95.9 117 84.6 90.2 71.4* 88.1 75.8
Minegishi et al. 83	2022	No	Neoplastic (including SSLs) vs. non-neoplastic	NBI	395 diminutive polyps (259 AD, 25 SSL, 111 NNP) of which at least 126 in RS (exact amount unspecified)	16 endoscopists (11 board certified experts with ≥ 5 years experience, 5 non-experts)	Success rate CADx (%) HC diagnosis (%) Number of polyps Accuracy (%) Sensitivity (%) Specificity (%) PPV (%) NPV (%)	94.2 N/A 372 84.4 93.3 61.5 N/A N/A	N/A	N/A N/A 372 85.5 94.4 62.5 N/A N/A	N/A	N/A 92.7 366 88.0 95.8 67.0 88.5 85.9	N/A N/A 126 85.7 95.2 76.2 80.0 94.1
Rondonotti et al. 84	2022	Yes	Adenomas vs. non-adenomas (including SSLs)	BLI	596 diminutive polyps (259 AD, 337 NAD), all in RS	18 endoscopists (9 expert endoscopists, 9 non-expert endoscopists ^†††^ )	Success rate CADx (%) HC diagnosis (%) Number of polyps Accuracy (%) Sensitivity (%) Specificity (%) PPV (%) NPV (%)	N/A	90.8 N/A 541 85.8 81.9 88.7 84.4 86.7	N/A	N/A 90.6 540 88.7 88.6 88.8 86.1 90.1	N/A	N/A 92.3 550 88.4 88.6 88.1 85.1 91.0
CADx, computer-aided diagnosis; SSL, sessile serrated lesion; NBI, narrow band imaging; AD, adenomas; NAD, non-adenomas; RS, rectosigmoid; N/A, not available; HC, high confidence; PPV, positive predictive value; NPV, negative predictive value; NP, neoplastic polyp; NNP, non-neoplastic polyp; WLE, white light endoscopy; HP, hyperplastic polyp; BCSP, bowel cancer screening program; BLI, blue light imaging. * Significant difference compared to CADx performance ( *P* < 0.05). ^†^ If available, performance with inclusion of only high-confidence diagnoses reported. ^‡^ Only concordance between optical diagnosis by endoscopists and CADx system diagnosis reported (97.5%). ^§^ Reported results concern 'worst-case scenario' results as reported in study (9 polyps for which CADx system diagnosis was not possible were treated as either false-positive or false-negative). ^¶^ CADx performance benchmarked to endoscopists in separate (non-real-time) test set, hence not reported within table. ^††^ High-confidence diagnosis cut-off threshold: 70%. ^‡‡^ High-confidence diagnosis cut-off threshold: 80%. ^§§^ Within this study, CADx trained and tested for white light endoscopy. However, endoscopists used virtual chromoendoscopy for optical diagnosis and were not blinded for the CADx diagnosis. While unblinded to CADx diagnosis, endoscopist performance is reported as CADx-assisted performance. ^¶¶^ High-confidence diagnosis cut-off threshold: 50%. ^†††^ Experts endoscopists followed a dedicated training program, underwent periodic auditing and monitoring and performed optical diagnosis on a regular basis. Non-experts were endoscopists that did not fulfill these criteria.													

To facilitate implementation of optical diagnosis strategies in daily practice, the Preservation and Incorporation of Valuable Endoscopic Innovations (PIVI) initiative 23 and Simple Optical Diagnosis Accuracy (SODA) 24 competence standards have been described. In Table 4 , results of described clinical trials are evaluated along the lines of these standards. While most CADx systems were able to meet several of the performance thresholds, none of them met all thresholds. This does however also relate to the fact that only two studies reported all required parameters 82 84 . Besides, an important issue to address is that, according to PIVI and SODA standards, only high-confidence (HC) diagnoses should be used to calculate performance parameters 23 24 . Nonetheless, within most studies differentiation between high- and low-confidence CADx system diagnoses was either not described 77 78 81 84 , or results with and without inclusion of low-confidence diagnoses were not separately reported 79 80 83 . Moreover, a standard HC diagnosis threshold cut-off for CADx systems is lacking (i.e. threshold concerning the minimum degree of certainty that an algorithm requires to consider an output a HC diagnosis). This results in CADx systems adopting different HC diagnosis threshold cut-offs 79 80 82 , making reliable comparison and evaluation impossible.

**Table:** **Table 4** Performance of deep learning-based computer-aided diagnosis systems for endoscopic differentiation of neoplastic from non-neoplastic polyps evaluated in the context of PIVI and SODA criteria.

	Threshold definition		Kominami et al. 77 ^a^	Mori et al. 78 ^*^	Barua et al. 79	Garcia-Rodríguez et al. 80 ^‡^	Hassan et al. 81 ^*^	Houwen et al. 82 ^§^	Minegishi et al. 83 ^*^	Rondonotti et al. 84 ^§^
PIVI-1	≥ 90% agreement with USMSTF or ESGE post-polyectomy guidelines	Agreement USMSTF guideline (%) Agreement ESGE guideline (%)	N/A ^¶^ N/A	N/A N/A	N/A N/A	N/A N/A	95.9 (yes) 95.6 (yes)	N/A 95.5 (yes)	90.1 (yes) 93.4 (yes)	92.1 (yes) 96.8 (yes)
PIVI-2	≥ 90% negative predictive value for neoplastic lesions in the rectosigmoid	NPV (%)	N/A ^**^	95.2 (yes)	N/A	N/A	97.6 (yes)	78.6 (no)	N/A	86.7 (no)
SODA-1	≥ 90% sensitivity and 80% specificity for high-confidence endoscopic characterization of colorectal neoplasia of 1–5 mm in the rectosigmoid	Sensitivity (% Specificity (%)	N/A N/A	93.3 (yes) 95.2 (yes)	N/A N/A	N/A N/A	82.0 (no) 93.2 (yes)	91.7 (yes) 37.8 (no)	N/A N/A	81.9 (no) 88.7 (yes)
SODA-2	≥ 80% sensitivity and 80% specificity for high-confidence endoscopic characterization of colorectal neoplasia of 1–5 mm	Sensitivity (% Specificity (%)	93.0 (yes) 93.3 (yes)	92.7 (yes) 89.8 (yes)	N/A N/A	88.2 (yes) 61.1 (no)	82.0 (yes) 89.5 (yes)	89.4 (yes) 38.3 (no)	93.3 (yes) 61.5 (no)	81.9 (yes) ^††^ 88.7 (yes) ^††^
PIVI, Preservation and Incorporation of Valuable Endoscopic Innovations; SODA, Simple Optical Diagnosis Accuracy; USMSTF, United States Preventive Services Taskforce; ESGE, European Society of Gastrointestinal Endoscopy; N/A, not available; NPV, negative predictive value. *No specific information concerning differentiation between low- and high-confidence diagnoses by the CADx system reported within this study. Reported results based on all included polyps or all polyps for which a CADx system diagnosis was available. †Stand-alone CADx performance not reported within this study. ‡Differentiation between low- and high-confidence diagnoses by the CADx system reported within this study (threshold cut-off value reported in Table 3 ), but results for high-confidence CADx system diagnoses only not available. Reported results based on both low- and high-confidence CADx system diagnoses. §Differentiation between low- and high-confidence diagnoses by the CADx system reported within this study (threshold cut-off value reported in Table 3 ). Reported results based on high-confidence CADx system diagnoses only. ¶No guideline agreement reported for diminutive polyps specifically (guideline agreement for all included polyps: 92.7%). **No NPV reported for diminutive rectosigmoid polyps specifically (NPV for all detected diminutive polyps: 93.3%). ††Study only includes rectosigmoid polyps, hence similar to performance as reported for SODA-1 criterion.										

From clinical perspective, the fact that different studies managed sessile serrated lesions (SSLs) in different ways should also be addressed. While SSLs are estimated to make up 15–30% of CRC cases 85 and especially optical differentiation between SSLs (neoplastic) and hyperplastic polyps (non-neoplastic) is known to be challenging 86 , only two studies used a CADx system that was specifically trained for recognition of SSLs [82 83]. Besides, only three studies (partly) included SSLs within the neoplastic polyp group 79 82 83 , while others assigned SSLs to the non-neoplastic group 80 81 84 or excluded all SSLs 77 78 . Additional limitations relate to the fact that the number of included polyps was low in several studies, most studies were single center and only two studies involved ‘non-expert’ endoscopists 79 84 .

Despite remaining limitations and need for further optimization of system performances to reach PIVI and SODA thresholds, most CADx systems for differentiation of neoplastic and non-neoplastic lesions showed to be able to meet expert endoscopist performance in real-time clinical settings. In addition, a significant optical diagnosis learning curve for ‘non-expert’ endoscopists was illustrated 84 . In the last place, CADx showed the potential to increase the proportion of HC diagnoses by endoscopists compared to unaided optical diagnosis 79 82 . This is crucial to establish a reduction in unnecessary polypectomies and pathological assessments 23 24 . Hence, with the commercial availability of some of the evaluated CADx systems 87 , first steps toward improved polyp assessment with use of CADx systems might be ahead.

### Differentiation between polyps with different degrees of dysplasia

Several studies assessing the feasibility of deep learning approaches for differentiation of polyps with different degrees of dysplasia (LGD versus HGD) are available ( **Table S1** ) 88 89 90 91 92 93 . This is relevant as lesions harboring HGD should ideally be resected en bloc 4 25 and may warrant shortened surveillance intervals 27 28 .

Reported accuracies in six identified studies ranged between 80.2 and 94.6% 88 89 90 91 92 93 . In three of these studies, the CADx systems outperformed endoscopists with different levels of experience 88 91 93 . However, none of the proposed systems was evaluated in a real-time clinical setting and most studies also included lesions other than lesions with LGD or HGD.

Because the prevalence of HGD in diminutive polyps is low 94 95 96 , the additional value of these systems for optical diagnosis strategies is uncertain. Nonetheless, they may be useful for development of algorithms for purposes such as identification of areas with advanced dysplasia in larger lesions. Moreover, while most algorithms are also trained for recognition of adenocarcinoma, these algorithms might be useful to address clinical challenges such as endoscopic recognition of T1 CRCs 97 98 .

### Differentiation between superficial and deep invasive lesions

In case of a suspected CRC, the choice and feasibility of en bloc resection methods depends on the depth of invasion 25 26 . Nonetheless, imaging modalities to accurately determine lesion invasion depth are lacking. Hence, differentiation of lesions with and without DSI is mostly done based on endoscopic identification of specific morphological polyp features 20 21 71 72 and surface characteristics 99 100 that are known to be associated with DSI. However, this endoscopic differentiation is known to be challenging 20 21 22 .

Deep learning approaches for differentiation of lesions with and without DSI have been proposed in several studies ( Table 5 ) 101 102 103 104 105 106 107 108 . Identified studies reported accuracies ranging between 81.2% and 94.1% 101 102 103 104 105 106 107 108 . Some of these studies benchmarked CADx system performance to performance of endoscopists with variable degrees of experience 102 104 105 106 107 108 . In a few studies, the CADx system outperformed one or more of the novices and trainees 104 105 106 108 . In addition, one study illustrated that diagnostic accuracy of endoscopists improved with assistance of a CADx system 108 . However, in none of the studies the CADx system was able to significantly outperform experienced or expert endoscopists.

**Table:** **Table 5** Overview of studies describing deep learning approaches for endoscopic differentiation of lesions with and without deep submucosal invasion.

	Year	Included lesions within test set	Imaging modality	Datasets†	Endoscopist comparison group(s) (experience)		Results			
							CADx	Endoscopists		
								G1	G2	G3
Takeda et al. 101	2017	Non-invasive (AD) vs. invasive (CRC with DSI)	Endocytoscopy	Training set: 5543 images Test set: 200 images	N/A	Accuracy (%) Sensitivity (%) Specificity (%) PPV (%) NPV (%)	94.1 ^‡^ 89.4 ^‡^ 98.9 ^‡^ 98.8 ^‡^ 90.1 ^‡^	N/A	N/A	N/A
Tamai et al. 102	2017	Non-invasive (HPs, AD, CRC without DSI) vs. invasive (CRC with DSI)	Magnified NBI	Training set: N/A Test set: 121 images	Group 1: Two experienced endoscopists (experience undefined)	Accuracy (%) Sensitivity (%) Specificity (%) PPV (%) NPV (%)	88.4 55.0 95.0 68.8 91.4	91.7 70.0 96.1 77.8 94.2	N/A	N/A
Ito et al. 103	2019	Non-invasive (CRC without DSI) vs. invasive (CRC with DSI)	WLE	Training set: 9,942 images Test set: 5,022 images	N/A	Accuracy (%) Sensitivity (%) Specificity (%) PPV (%) NPV (%)	81.2 67.5 89.0 N/A N/A	N/A	N/A	N/A
Lui et al. 104	2019	Non-invasive (SSL, AD, CRC without DSI) vs. invasive (CRC with DSI)	WLE, NBI	Training set: 8000 images Test set: 567 images	Group 1: One expert (> 2000 colonoscopies) Group 2: Two trainees (> 500 colonoscopies, NBI training)	Accuracy (%) ^§^ Sensitivity (%) ^§^ Specificity (%) ^§^ PPV (%) ^§^ NPV (%) ^§^	85.5 ^¶^ 88.2 ^¶^ 77.9 ^¶^ 92.1 ^¶^ 69.3 ^¶^	86.4 91.8 52.6 92.4 50.6	71.0 73.9 61.8 85.7 47.2	N/A
Nakajima et al. 105	2020	Non-invasive (CRC without DSI) vs. invasive (CRC with DSI)	WLE	Training set: 1839 image Test set: 78 images	Group 1: Two experts (> 5000 colonoscopies) Group 2: Two trainees (< 500 colonoscopies) Group 3: Two novices (15 minutes education by case study, < 6 months residency)	Accuracy (%) ^**^ Sensitivity (%) ^**^ Specificity (%) ^**^ PPV (%) ^**^ NPV (%) ^**^	84.1 81.0 87.0 85.0 83.3	92.0 85.7 97.8 97.3 88.2	71.5 83.3 60.9 65.4 83.3	65.9 100 34.8 58.7 100
Lu et al. 106	2021	Non-invasive (CRC without DSI) vs. invasive (CRC with DSI)	WLE, magnified and non-magnified NBI/BLI	Training set: 21,433 images Test set: 168 images	Group 1: Three experts (> 10 years’ experience) Group 2: Five seniors (> 5 years’ experience) Group 3: Seven juniors (> 1 year experience)	Accuracy (%) ^††^ Sensitivity (%) ^††^ Specificity (%) ^††^ PPV (%) ^††^ NPV (%) ^††^	88.1 92.5 85.6 79.0 95.0	84.1 64.2 95.8 91.4 82.1	84.1 64.2 95.8 91.4 82.1	76.7 62.3 85.4 76.1 80.3
Luo et al. 107	2021	Non-invasive (AD, CRC without DSI) vs. invasive (CRC with DSI)	WLE	Training set: 7734 images Test set: 1634 images	Group 1: Two experts (> 200 ESD cases), six experienced endoscopists (> 3000 colonoscopies, > 200 EMR cases, > 30 ESD cases)	Accuracy (%) Sensitivity (%) Specificity (%) PPV (%) NPV (%)	91.1 91.2 91.0 86.7 93.7	92.6 88.4 95.5 93.2 92.2	N/A	N/A
Tokunaga et al. 108	2021	Non-invasive (AD, CRC without DSI) vs. invasive (CRC without DSI)	WLE	Training set: 2751 images Test set: 691 images	Group 1: Two experts (> 2000 colonoscopies + 500 EMRs/ESDs) Group 2: Two trainees (< 500 colonoscopies)	Accuracy (%) Sensitivity (%) Specificity (%) PPV (%) ^‡‡^ NPV (%) ^‡‡^	90.3 96.7 75.0 90.2 90.5	89.4 96.5 72.5 89.4 89.7	84.9* 92.1* 67.6* 87.2 78.2	N/A
CADx, computer-aided diagnosis; G1, group 1; G2, group 2; G3, group 3; AD, adenoma; CRC, colorectal cancer; DSI, deep submucosal invasion; N/A, not available; PPV, positive predictive value; NPV, negative predictive value; HP, hyperplastic polyp; NBI, narrow band imaging; WLE, white light endoscopy; SSL, sessile serrated lesion; BLI, blue light imaging; EMRs, endoscopic mucosal resections; ESDs, endoscopic submucosal dissections. *Significant difference compared to CADx performance (P < 0.05). †Data used for internal validation is reported as part of the training set. ‡Reported results concern results including both high- and low-confidence diagnoses. Performance for high-confidence diagnoses only (72.0% of included polyps): accuracy 99.3%, sensitivity 98.1%, specificity 100%, PPV 100%, NPV 98.8%. §Reported results for endoscopists in group 2 (trainees) concern calculated means. No P value for comparison of mean to CADx system performance available. On individual basis, the CADx system significantly outperformed one of the trainee endoscopists on accuracy, sensitivity, and NPV. The other trainee endoscopist was significantly outperformed by the CADx system on accuracy, specificity, and PPV. ¶Reported results concern average performance of CADx system for both narrow band imaging and white light endoscopy images. **Reported results for endoscopists concern calculated means for separate endoscopists groups. No P value for comparison of means to CADx system performance available. On individual basis, the CADx system reached significantly higher accuracy than one of the trainee endoscopists and one of the novice endoscopists. CADx system reached significantly higher specificity than both novice endoscopists. Both novice endoscopists and one trainee endoscopist reached significantly higher sensitivity than the CADx system. ††Reported results for endoscopists concern calculated means for separate endoscopist groups. No P value for comparison of means to CADx system performance available. On individual basis, CADx system significantly outperformed five of seven junior endoscopists on overall performance, but none of the senior or expert endoscopists. ‡‡No significance levels for difference between endoscopist and CADx system performance available for NPV and PPV.										

Although these results seem promising, they should be carefully interpreted. Firstly, none of the systems was validated in a real-time clinical setting. Moreover, CADx systems were trained and validated using different imaging modalities, with two studies showing that performance may differ per imaging modality 104 106 . Besides, the datasets considerably differed in both size and composition. Only three studies reported CADx systems that were tested on datasets consisting of CRCs only (both with and without DSI) 103 105 106 while other studies also included benign lesions in the non-DSI group 101 102 104 107 108 .

With recent introduction of new endoscopic resection methods, possibilities for local resection for lesions with DSI seem to be increasing. As a result, it could be debated whether optical diagnosis should not be adapted to also differentiate lesions with different degrees of DSI 109 . This might also have implications for future development of CADx systems designed for assessment of CRC invasion depth. However, clinical validation of currently available CADx systems is warranted first.

## Discussion

Over the past decade, advances in AI and computer science have led to an exponential increase in studies on computer-aided diagnosis of colorectal polyps. As outlined within this review, the most substantial developments in the field of computer-aided polyp diagnosis involve CADx systems for differentiation between neoplastic and non-neoplastic lesions. Several studies have demonstrated potential of such systems to meet expert performance levels in real-time clinical settings. Developmental processes of computer systems that are able to provide real-time feedback to endoscopists on polyp characteristics such as size, location, degree of dysplasia and invasion depth are still in preliminary phases. Future studies should mainly focus on prospective clinical validation of these systems. Besides, feasibility of CADx systems for specific assessment of polyp morphology has yet to be explored.

Adopting computer systems for colorectal polyp assessment in daily practice might yield several benefits. Primarily, if these systems are trained with high quality expert-annotated data, they could possibly serve as an objective, expert-level second observer that is not prone to human factors such as fatigue, distraction or subjectivity. Especially for less experienced endoscopists, this could provide opportunities to optimize accuracy of polyp assessments, thereby possibly improving clinical outcomes and reducing patient burden and costs. In addition, availability of computer systems able to assess independent polyp characteristics could provide possibilities for automated polyp description for endoscopy reports 75 . When combined with algorithms for purposes such as recognition of resection methods 110 , this might significantly ease administrative burdens for endoscopists. In the last place, optimizing accuracy of endoscopic assessment of different polyp characteristics could aid in development of more trustworthy clinical decision-making algorithms or prediction models involving specific polyp characteristics 111 112 113 .

On the other hand, clinicians should also be aware of the limitations and potential disadvantages of computer-aided polyp diagnosis. Especially systems based on machine learning architectures are highly dependent on the training data used. While these systems are often trained with human-annotated data, these systems are not likely to outperform experts on a regular basis. Therefore, clinicians should be aware that these systems are not flawless. In addition, system performance is also dependent on what is shown by the endoscopist: the quality of the images provided to the computer system during endoscopies might differ between endoscopists, possibly influencing system performance and feasibility 82 . Moreover, it can be hypothesized that regular CADx system-assisted colonoscopy might eventually lead to a certain degree of user-dependency.

There are also several more general issues to be addressed when considering the future perspectives of CADx systems in endoscopy practice. In the first place, insights into the cost-effectiveness of CADx systems are still scarce. Although it is suggested that CADx could potentially lead to a 11% reduction of average colonoscopy costs 114 , figures concerning actual cost reduction due to use of CADx systems in different countries and clinical settings are still lacking. Second, there might be limitations concerning the technical integration of CADx systems in different endoscopy suites and settings, while most systems have unique hardware and software requirements and are not simply compatible with all regularly used endoscopy devices. Third, the sentiment of physicians toward AI and computer-aided diagnosis should be taken in consideration: increased costs, operator dependency and increased procedural time are common concerns among physicians 115 . Moreover, basic technical knowledge on topics such as machine learning is warranted to be able to critically appraise available literature on the topic of computer-aided diagnosis approaches and appraise the possible technical biases inherent to available systems. Due to the novelty of AI and computer-aided diagnosis, most clinicians will likely lack this knowledge. Therefore, specific education and training will be needed to increase its feasibility.

Despite the various limitations and uncertainties, it should be emphasized that computer-aided diagnosis has only been a topic of interest within the field of gastrointestinal endoscopy for a little over ten years. Hence, especially in the context of the rapidly increasing amounts of research on this topic, toward the future computer-aided diagnosis will likely take a more prominent role in daily endoscopy practice. On one hand this relates to the fact that (technical) innovations in upcoming years will likely aid in improving accuracy of existing CADx systems, while there are also still numerous purposes for which possibilities of computer-aided diagnosis is yet to be explored. In example, besides computer systems that could aid endoscopists in assessment of polyp morphology, systems for purposes such as suggestion of appropriate polyp resection method or assessment of completeness of resection might yield significant clinical potential.

The strength of this review is that, to the best of our knowledge, this is the first review to provide such a broad overview of available studies on computer-aided diagnosis of all polyp characteristics essential for clinical decision making. However, in the context of the extensive scope of the aim of this review, we decided to comply to a narrative rather than a systematic review approach. While this might have resulted in accidental miss of relevant publications, this can be considered a limitation.

## Conclusions

To conclude, with recent breakthroughs in the field of AI and computer science, a major increase in research on the topic of computer-aided colorectal polyp assessment is seen. With commercial availability of CADx systems for differentiation between neoplastic and non-neoplastic polyps, first steps toward improved endoscopy quality with use of CADx systems in daily practice might be ahead. However, optimization of performance is still required to ensure that these CADx systems meet all performance thresholds. Besides, toward the future, further innovation, exploration and clinical validation of computer-aided diagnosis approaches for diagnosis of other polyp characteristics is required for realization of complete computer-aided polyp assessment.

## Supporting information

Supplementary material Zusatzmaterial
