# Correction: Advances in artificial intelligence and computer science for computer-aided diagnosis of colorectal polyps: current status

**DOI:** 10.1055/a-2159-4772

**Published:** 2023-08-24

**Authors:** Querijn NE van Bokhorst, Britt BSL Houwen, Yark Hazewinkel, Paul Fockens, Evelien Dekker

**Affiliations:** 1 26066 Department of Gastroenterology and Hepatology, Amsterdam University Medical Centers, location Academic Medical Center, Amsterdam, the Netherlands; 2 571165 Amsterdam Gastroenterology Endocrinology Metabolism, Amsterdam, the Netherlands; 3 3913 Department of Gastroenterology and Hepatology, Tergooi Medical Center, Hilversum, the Netherlands



In the above-mentioned article the previously missing abstract was added. This was corrected in the online version on 25.08.2023.

## Abstract

Colonoscopy is considered the gold standard for detection of colorectal cancer and its precursor lesions. However, colonoscopy outcomes may differ depending on the endoscopist performing the procedure. Among others, this relates to differences in ability of endoscopists to accurately assess polyp characteristics that are essential for clinical decision making. These characteristics concern polyp location, size and morphology, as well as several histological polyp features that can be predicted based on polyp phenotype. Polyp assessment with aid of computer-aided diagnosis (CADx) systems might provide opportunities to optimize general polyp assessment accuracy. However, a broad overview of available studies concerning performance of CADx systems for diagnosis of different polyp characteristics and histological features is lacking. Hence, within this narrative review we aimed to provide such an overview. We highlight that most significant advancements in the field of computer-aided polyp assessment involve systems for optical differentiation between neoplastic and non-neoplastic lesions, with several studies showing the ability of such systems to perform at expert levels in real-time clinical settings. With commercial availability of some of these systems, first steps towards improved endoscopy quality with use of CADx systems in daily practice might be ahead. However, development of CADx systems for assessment of polyp characteristics size and location, as well as prediction of degree of dysplasia and invasion depth, are still in more preliminary stages while evaluation of these systems in real-time clinical settings is still warranted. Moreover, computer-aided diagnosis of polyp morphology is a field yet to be explored.

