# Supplementary material for: Advances in artificial intelligence and computer science for computer-aided diagnosis of colorectal polyps: current status
Source: Endosc Int Open. 2023 Aug 16;11(8):E752–67. doi: 10.1055/a-2098-1999 (PMC10431975; doi:10.1055/a-2098-1999)
Supplement: Supplementary file 1 — Supplementary material [file 10-1055-a-2098-1999_21027843.pdf.pdf]

Supplementary material

Table S1 Overview of studies describing deep learning approaches for endoscopic differentiation of polyps with different grades of dysplasia (low grade dysplasia versus high grade dyplasia)

	Year	Classification groups	Imaging modality	Datasets[a]	Endoscopist comparison group(s) (experience)	Results	CADx	Endoscopists G1	Endoscopists G2
**Choi et al. [1]**	2020	- Normal (no adenoma)<br>- Adenoma with LGD<br>- Adenoma with HGD<br>- Adenocarcinoma	WLE	Training set: 3000 images<br><br>Test set: 200 images	Group 1: 4 ‘experts’ (>5 years)<br>Group 2: 6 ‘trainees’ (<2 years)	Accuracy (%)[b]<br>Sensitivity (%)[b]<br>Specificity (%)[b]<br>PPV (%)[b]<br>NPV (%)[b]	90.0<br>87.2<br>96.7<br>87.6<br>96.8	85.0<br>85.0<br>95.0<br>85.7<br>95.1	77.9<br>78.9<br>92.6<br>78.4<br>92.8
**Yang et al. [2]**	2020	- Non-advanced lesion (non-neoplastic lesions and TA with LGD)<br>- Advanced lesions (adenoma with HGD and CRC stage T1- T4)	WLE	Training set: 3828 images<br><br>Test set: 240 images	Group 1: 2 ‘experts’ (>5 years), 1 ‘trainee’ (< 6 months)	Accuracy (%)[c]<br>Sensitivity (%)[c]<br>Specificity (%)[c]<br>PPV (%)[c]<br>NPV (%)[c]	94.6<br>51.5<br>96.7<br>43.0<br>97.6	96.6<br>72.7<br>97.7<br>65.9<br>98.7	N/A
**Zorron Cheng Tao Pu et al. [3]**	2020	- HP<br>- SSA/P<br>- Adenoma with LGD<br>-Adenoma with HGD/(tubulo)villous histology/superficial cancer<br>- Invasive cancer	Magnified NBI, BLI	Training set: 1235 images<br><br>Test set 1: 69 images	N/A	Accuracy (%)<br>Sensitivity (%)<br>Specificity (%)<br>PPV (%)<br>NPV (%)	N/A[d]	N/A	N/A
**Choi et al. [4]**	2021	- Normal (no adenoma)<br>- Adenoma with LGD<br>- Adenoma with HGD<br>- Adenocarcinoma	WLE	Training set: 3000 images<br><br>Test set: 200 images	Group 1: 4 ‘experts’ (>5 years)<br>Group 2: 6 ‘trainees’ (<2 years)	Accuracy (%)[b]<br>Sensitivity (%)[b]<br>Specificity (%)[b]<br>PPV (%)[b]<br>NPV (%)[b]	N/A<br>77.3<br>92.4<br>77.2<br>92.6	67.4<br>72.4<br>90.6<br>71.4<br>90.9	N/A<br>62.5<br>86.5<br>61.9<br>87.1
**Gong et al. [5]**[e]	2022	- Non-neoplasm<br>- Tubular adenoma with or without LGD<br>- Adenoma with HGD or early CRC<br>- Advanced CRC	WLE	Training set: 3828 images<br><br>Test set: 3818 images	N/A	Accuracy (%)<br>Precision (%)<br>Recall (%)<br>F1 score (%)	80.2<br>78.5<br>78.8<br>78.6	N/A	N/A
**Meng et al. [6]**	2022	- Adenoma with LGD<br>- Adenoma with HGD	WLE	Training set: 4884 images<br><br>Test set: 365 images	Group 1: 2 ‘experts’ (>10 years)	Accuracy (%)<br>Sensitivity (%)<br>Specificity (%)<br>PPV (%)<br>NPV (%)	88.2<br>85.4<br>89.8<br>82.2<br>91.7	71.5*<br>70.7*<br>71.9*<br>58.3*<br>81.6*	N/A

Supplementary material

CADx, computer-aided diagnosis; G1, group 1; G2, group 2; LGD, low grade dyplasia; HGD, high grade dysplasia; WLE, white light endoscopy; PPV, positive predictive value; NPV, negative predictive value; TA, tubular adenoma; CRC, colorectal cancer; N/A, not available; HP, hyperplastic polyp; SSA/P, sessile serrated adenoma/polyp; NBI, narrow band imaging; BLI, blue light imaging

[a] Data used for internal validation is reported as part of the training set;
[b] No statistical analysis of differences between CADx system performance and performance by endoscopists reported;
[c] Reported values for endoscopists concern calculated means. The best performing endoscopist outperformed the CADx system on accuracy, sensitivity and PPV. CADx system performance was similar to that of the other two endoscopists;
[d] Only area under the curve (AUC) reported: 84.5% for NBI, 90.3% for BLI;
[e] Multiple test sets and deep learning models described within study. Results for model with highest accuracy reported.

REFERENCES

1. Choi K, Choi SJ, Kim ES. Computer-Aided Diagonosis for Colorectal Cancer using Deep Learning with Visual Explanations. In, 2020 42nd Annual International Conference of the IEEE Engineering in Medicine & Biology Society (EMBC); 2020: 1156-1159. doi:10.1109/EMBC44109.2020.9176653
2. Yang YJ, Cho BJ, Lee MJ et al. Automated Classification of Colorectal Neoplasms in White-Light Colonoscopy Images via Deep Learning. J Clin Med 2020; 9. doi:10.3390/jcm9051593
3. Zorron Cheng Tao Pu L, Maicas G, Tian Y et al. Computer-aided diagnosis for characterization of colorectal lesions: comprehensive software that includes differentiation of serrated lesions. Gastrointest Endosc 2020; 92: 891-899. doi:10.1016/j.gie.2020.02.042
4. Choi SJ, Kim ES, Choi K. Prediction of the histology of colorectal neoplasm in white light colonoscopic images using deep learning algorithms. Sci Rep 2021; 11: 5311. doi:10.1038/s41598-021-84299-2
5. Gong EJ, Bang CS, Lee JJ et al. No-Code Platform-Based Deep-Learning Models for Prediction of Colorectal Polyp Histology from White-Light Endoscopy Images: Development and Performance Verification. J Pers Med 2022; 12. doi:10.3390/jpm12060963
6. Meng S, Zheng Y, Wang W et al. A computer-aided diagnosis system using white-light endoscopy for the prediction of conventional adenoma with high grade dysplasia. Dig Liver Dis 2022. doi:10.1016/j.dld.2021.12.016. doi:10.1016/j.dld.2021.12.016
